# Gallium-68-somatostatin receptor PET/CT parameters as potential prognosticators for clinical time to progression after peptide receptor radionuclide therapy: a cohort study

**DOI:** 10.1186/s41824-021-00116-z

**Published:** 2021-12-09

**Authors:** Sander C. Ebbers, Muriël Heimgartner, Maarten W. Barentsz, Rachel S. van Leeuwaarde, Mark J. C. van Treijen, Marnix M. E. G. Lam, Arthur J. A. T. Braat

**Affiliations:** 1 Department of Radiology and Nuclear Medicine, University Medical Center Utrecht, Heidelberglaan 100, 3584 CX Utrecht, The Netherlands; 2 Department of Endocrine Oncology, University Medical Center Utrecht, Heidelberglaan 100, 3584 CX Utrecht, The Netherlands

**Keywords:** PRRT, [^68^Ga]Ga-DOTA-TOC PET/CT, Time-to-new treatment, Progression-free survival, PET-based response

## Abstract

**Background:**

Early [^68^Ga]Ga-DOTA-TOC PET/CT imaging after peptide receptor radionuclide therapy (PRRT) in neuroendocrine neoplasm patients is often used as a prognosticator for survival, but lacks validity. This study investigates the prognostic value of changes in PET parameters after PRRT.

**Methods:**

Baseline and follow-up [^68^Ga]Ga-DOTA-TOC PET/CT scans of all patients treated with PRRT were delineated automatically. Total lesion somatostatin receptor expression (TL-SSTR) and somatostatin receptor expressing tumor volume (SSTR-TV) were used as covariates in Cox proportional hazard models to predict time-to-new treatment.

**Results:**

In twenty patients, median time-to-new treatment was 19.3 months (range [3.8; 36.2]). Absolute and percentual changes in both PET parameters were not associated with time-to-new treatment. A significant relation between independent baseline and follow-up SSTR-TV and follow-up TL-SSTR, and time-to-new treatment was identified.

**Conclusions:**

Automatically derived [^68^Ga]Ga-DOTA-TOC PET/CT parameters are easy to acquire and may be of prognostic value after completing PRRT. Acquiring SSTR-TV or TL-SSTR parameters at baseline and during follow-up can be of value in identifying a patient’s prognosis.

## Key points

- *Question* Do changes in SSTR-PET/CT predict time-to-new-treatment after PRRT?
- *Pertinet findings* In patients treated with four cycles of PRRT, the total somatostatin-receptor expressing tumor volume and the total somatostatin receptor expression were calculated at baseline and after three months follow-up. Both baseline and follow-up tumor volume was significantly associated with early need for further treatment, while there was no association between the percentual or absolute change in these parameters and time-to-new-treatment.
- *Implications for patient care* SSTR-PET/CT based parameters are easy to acquire and seem to have prognostic value before and after PRRT in predicting the time to new treatment.

## Background

Peptide receptor radionuclide therapy (PRRT) with [^177^Lu]Lu-DOTA-TATE currently is the recommended treatment in case of disease progression in patients with metastasized grade I/II well-differentiated neuroendocrine neoplasms (NEN) that are treated with somatostatin analogs (SSA) (Kwekkeboom et al. 2009; Strosberg et al. 2017; Toriihara et al. 2019; Bushnell and Bodeker 2020; Starr et al. 2020). Treatment with PRRT using [^177^Lu]Lu-DOTA-TATE provides significant prolonged progression-free survival (PFS) and overall survival (OS), and symptom reduction, compared to high dose long acting SSA (Strosberg et al. 2017, 2018; Gabriel et al. 2019; Saravana-Bawan et al. 2019; Brabander et al. 2017). In the NETTER-1 trial, the PFS rate after 20 months increased from 10.8% (SSA group) to 65.2% (PRRT group). Multiple studies also showed potential benefit for patients with high-grade NEN in terms of OS, progression-free survival (PFS), and objective response (Carlsen et al. 2019; Zhang et al. 2019).

Despite the significant prolongation of OS and PFS, the outcome after PRRT varies among patients. In line with most oncological trials, the NETTER-1 used response evaluation criteria in solid tumors (RECIST 1.1), even though the response evaluation system with best prognostic performance is under debate (Vliet et al. 2013; Solis-Hernandez et al. 2019). Clinical response to PRRT is often monitored using ^68^Ga-somatostatin receptor targeted (^68^Ga-SSTR) PET/CT. A decrease in total tumor volume and total somatostatin receptor expression can frequently be observed on ^68^Ga-SSTR PET/CT, shortly after PRRT (Gabriel et al. 2009). However, no validation studies have been performed on the predictive value of ^68^Ga-SSTR PET/CT on enduring response. This study aims to investigate if the change in somatostatin receptor expression on ^68^Ga-SSTR PET/CT can be used to predict the time to initiation of new treatment after four cycles of PRRT.

## Materials and methods

### Patients

This cohort study included consecutive patients treated with PRRT using [^177^Lu]Lu-(HA-)DOTA-TATE in the University Medical Center Utrecht, The Netherlands. Eligibility criteria for treatment were grade I, II, or III well-differentiated NEN, somatostatin receptor expressing tumors (i.e., visually higher uptake of [^68^Ga]Ga-DOTA-TOC in tumors than in healthy liver tissue on PET/CT) and progressive disease for which no curative options were available. Patients were included in this study if all four cycles of PRRT were completed and if a [^68^Ga]Ga-DOTA-TOC PET/CT was available at baseline and within three months after the last cycle of PRRT. Patients with neuroendocrine carcinoma were excluded. Functional NEN was defined as having carcinoid symptoms (e.g., flushing, diarrhea). Treatment protocols were in concordance with the guidelines proposed by the European Neuroendocrine Tumor Society (ENETS) (Kwekkeboom et al. 2009).

### Study procedures

All [^68^Ga]Ga-DOTA-TOC PET/CT images were acquired on a Siemens Biograph mCT time-of-flight system (Siemens, Erlangen, Germany; injected activity 1.5–2.0 MBq/kg, acquisition time 3 min per bed position with approximately 43% overlap, reconstruction using 4 iterations with 21 subsets and a 5 mm full width at half maximum Gaussian post-reconstruction filter, reconstructed voxel size 4.1 × 4.1 × 1.5 mm^3^). In order to evaluate early response on ^68^Ga-DOTA-TOC imaging, the baseline and post-treatment scans were analyzed for changes in total somatostatin receptor expression and total tumor volume. Volumes of interest (VOI’s) were drawn semi-automatically using Syngo.Via (Siemens, Erlangen, Germany), after placement of a spherical reference VOI (3 cm in diameter, according to PERCIST guidelines) in healthy liver tissue. As a threshold, 1.5 times the standardized uptake value + 2 standard deviations of the healthy liver tissue VOI was used. Segmented regions with physiological uptake of [^68^Ga]Ga-DOTA-TOC were then manually removed (e.g., spleen, liver, pituitary, etc.). The final measures of uptake were total lesion somatostatin receptor expression (TL-SSTR) and somatostatin receptor expressing tumor volume (SSTR-TV), both variants of total lesion glycolysis (TLG) and metabolic tumor volume (MTV), as derived from the PERCIST criteria for [^18^F]FDG PET/CT (Wahl et al. 2009). SSTR-TV is equivalent to MTV, and was calculated by adding the volumes of each delineated tumor (cm^3^), thereby indicating total tumor burden in the patient. TL-SSTR is equivalent to TLG, and was calculated by multiplying the mean uptake per tumor by its corresponding SSTR-TV, and adding these within the patient (SUV-lbm × cm^3^).

### Outcomes

A proxy for clinical or radiological progression, time to initiation of new therapy was chosen as primary outcome, being a more clinically relevant and objective endpoint. Patients who warranted a new treatment after PRRT due to progression were considered having the event, while patients who were lost-to-follow-up or who had enduring response at the time of analysis were censored. Follow-up treatment was initiated at the discretion of the treating physician, and could be based on clinical symptoms, PET/CT, contrast-enhanced CT, or MRI imaging. The primary analysis consisted of the relation between percentual changes in TL-SSTR and SSTR-TV between baseline and three months post-treatment, and time-to-new treatment. As a secondary analysis, other factors were correlated to time-to-new treatment: absolute (difference in) TL-SSTR and SSTR-TV, tumor grade (as a categorical variable), primary tumor, functional tumor, baseline chromogranin A (CgA) levels, Eastern Cooperative Oncology Group (ECOG) performance, and hepatic tumor burden.

### Statistical analysis

Survival curves were analyzed using Kaplan–Meier analysis. For the primary and secondary analysis, predictors for time to progression were analyzed using Cox proportional hazard models (Cox-PH), from which hazard ratios (HR) and confidence intervals (CI) were extracted. An explorative analysis was performed to identify added value of parameters (i.e., absolute values and change of TL-SSTR and SSTR-TV). To facilitate plotting of continuous predictor variables in survival curves, continuous variables were cut to categorical variables at − 0.5 and 0.5 standard deviations. Change in TL-SSTR and SSTR-TV was calculated by dividing the difference between baseline and follow-up values by the baseline value. Raw and absolute differences in TL-SSTR and SSTR-TV were normalized to the mean prior to adding them as covariates to the Cox-PH model. Consequently, output of these models consists of HRs per standard deviation (SD). All Cox-PH models were checked for proportional hazards assumption. All analyses were done in R version 4.0.2. Differences were considered statistically significant with a two-sided *p* value of 0.05.

## Results

**Fig. 1:**
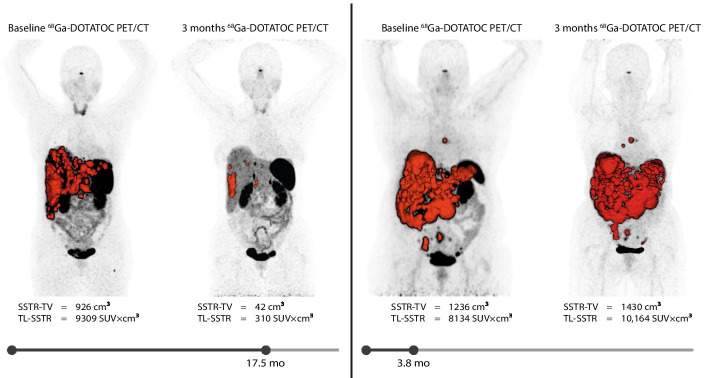
Example cases. Two patients with a low (left; SSTR-TV_baseline_ change -95% and TL-SSTR_baseline_ change -97%) and high (right; SSTR-TV_baseline_ change 16% and TL-SSTR_baseline_ change 25%) residual tumor burden following PRRT. The automatically derived tumor VOI’s used to calculate SSTR-TV and TL-SSTR are visualized in red. The patient on the left (female, 51 yo) had a grade 2 tumor of unknown primary (Ki67 4–6%); the patient on the right (female, 57 yo) had a grade 1 small intestine neuroendocrine tumor (Ki67 2%). Both patients had > 25% liver tumor load. Consequently, the patient on the left had a long time-to-new treatment (17.5 months), while the patient on the right required additional treatment rather quickly, because of progressive disease under PRRT (new intrathoracic and intra-abdominal lymph node metastases)

Between September 2016 and December 2019, 44 NEN patients were treated with PRRT. Twenty-three completed all four cycles, and were eligible for inclusion. Twenty patients were included in the final analysis, as [^68^Ga]Ga-DOTA-TOC PET/CT was unavailable in two patients, and one patient’s baseline [^68^Ga]Ga-DOTA-TOC PET/CT scan was of insufficient quality: no spherical reference VOI could be reliably drawn, and hence, no uptake calculations could be made. 7/20 (35%) patients had a grade I NEN, 10/20 (50%) had a grade II NEN, and 3/20 (15%) had a grade III NEN (Ki-67 20%, 26%, and 50%; Table 1). All patients showed evidently higher uptake of [^68^Ga]Ga-DOTA-TOC in tumor tissue than in healthy liver tissue as visually scored using the Krenning score. Two example patients are shown in Fig. 1.

**Table 1:** Baseline characteristics

	N (%)
Number of patients	20
Age—median (IQR)	62.5 (58–74)
Male gender	11 (55%)
Years since diagnosis—median (IQR)	4.3 (1.5–11.8)
ECOG PS	
0	13 (65%)
1	7 (35%)
Functional tumor	7 (35%)
MEN1	4 (20%)
Hepatic tumor burden	
< 25%	14 (70%)
> 25%	6 (30%)
Baseline chromogranin A—median (IQR)	580 (183.5–1811)
Type of tumor	
Intestine	9 (45%)
Pancreas	4 (20%)
Insulinoma	2 (10%)
Gastrinoma	1 (5%)
Lung	1 (5%)
VIPoma	1 (5%)
Unknown	2 (10%)
WHO grade	
1	7 (35%)
2	10 (50%)
3	3 (15%)
Tissue involvement	
Liver	18 (90%)
Lymph nodes	12 (60%)
Bone	5 (25%)
Previous treatments	
SSA	17 (85%)
Surgery	9 (45%)
SIRT	4 (20%)
Chemotherapy	1 (5%)
External-beam radiotherapy	1 (5%)
Mean pre-SUV-lbm_liver_ (sd)	4.61 (± 2.24)
Mean post-SUV-lbm_liver_ (sd)	3.90 (± 1.79)

IQR: interquartile range; ECOG PS: Eastern Cooperative Oncology Group Performance Status; WHO: World Health Organization; SIRT: selective internal radiotherapy; SSA: somatostatin analogs

The main endpoint, median time-to-new treatment, was 19.3 months (range [3.8; 36.2]). In total, ten (50%) patients reached their primary endpoint. These patients were referred for additional treatment after PRRT because of radiological (8 patients) or clinical progression (2 patients). The decision to refer for additional treatment in one patient was based on the [^68^Ga]Ga-DOTA-TOC PET/CT used for uptake measurements within 3 months after follow-up. One patient, who was censored, progressed quickly, and died before any additional treatment could be initiated. The other nine patients were still being followed-up without requiring additional treatment at the time of analysis. No patients were lost-to-follow-up.

Mean baseline TL-SSTR and SSTR-TV were 5042 SUV-lbm × cm^3^ (95% CI [2161; 7923]) and 505 cm^3^ (95% CI [184; 826]), which reduced after treatment to 2742 SUV-lbm × cm^3^ (95% CI [827; 4656]) and 291 cm^3^ (95% CI [77; 505]). Both TL-SSTR and SSTR-TV after treatment were lower in all but two patients (18/20, *p* = 0.048 and *p* = 0.042). The two patients with an increase in TL-SSTR and SSTR-TV had a grade one and grade two small intestine NET with high (> 25%) and moderate (10–25%) liver tumor burden. Four patients had a reduction of < 30% in TL-SSTR, two had a reduction of 30–50% in TL-SSTR, five had a reduction of 50–80% in TL-SSTR, and seven patients had a reduction of > 80% in TL-SSTR. Furthermore, three patients had a reduction of < 30% in SSTR-TV, five had a reduction of 30–50% in SSTR-TV, six had a reduction of 50–80% in SSTR-TV, and four patients had a reduction of > 80% in SSTR-TV.

**Fig. 2:**
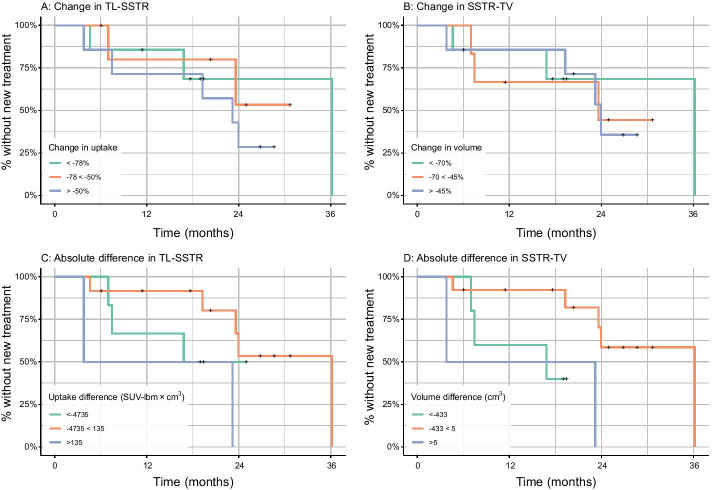
Changes in PET-parameters and time-to-new treatment. Effect of changes in TL-SSTR and SSTR-TV on the time-to-new treatment. Cutoff points were established by taking the − 0.5 and 0.5 standard deviations for visualization

The percentual change in TL-SSTR or SSTR-TV did not yield a difference in hazard rate for time-to-new treatment (*p* = 0.43 and *p* = 0.45, Fig. 2A, B). Hazard ratios (HR) per 100% increase in TL-SSTR or SSTR-TV were 1.24 (95% CI [0.76; 2.00]) and 1.32 (95% CI [0.69; 2.52]). The absolute differences in both parameters also were insignificant covariates (*p* = 0.52 and *p* = 0.32, Fig. 2C, D).

**Fig. 3:**
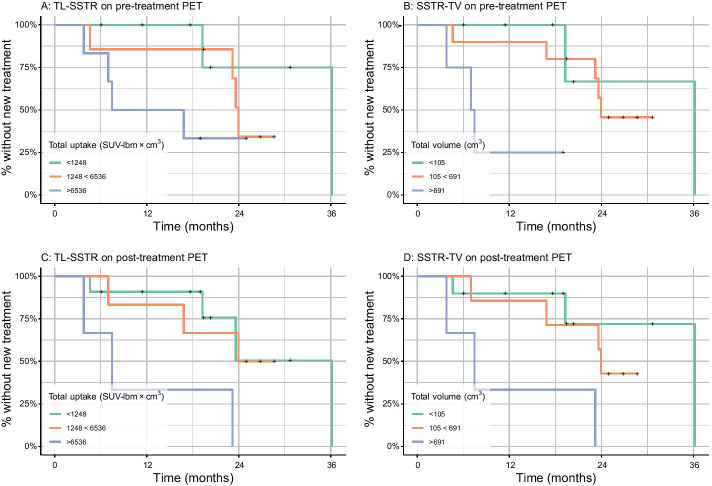
Baseline and follow-up PET-parameters and time-to-new treatment. Effect of absolute baseline and follow-up TL-SSTR and SSTR-TV on the time-to-new treatment. Cutoff points were established by taking the − 0.5 and 0.5 standard deviations for visualization. Only post-treatment SSTR-TV (D) was significantly associated with survival

Baseline and post-treatment SSTR-TV were independently associated with the hazard of time-to-new treatment (HR_baseline_ per SD 1.74 [1.10–2.75], *p* = 0.03 and HR_post-treatment_ per SD 3.03 [1.36–6.77], *p* = 0.01; Fig. 3B, D). In other words, a high tumor load resulted in a higher HR, both pre- and post-treatment. Adding both baseline and post-treatment SSTR-TV as covariates did not improve the model (*p* = 0.95). Post-treatment TL-SSTR as independent variable was also associated with the hazard of time-to-new treatment (HR_post-treatment_ per SD 2.26 [1.09–4.73], *p* = 0.04; Fig. 3C), while baseline TL-SSTR and both covariates in one model were nonsignificant in Cox-PH analysis (*p* = 0.06 and *p* = 0.37; Fig. 3A).

**Fig. 4:**
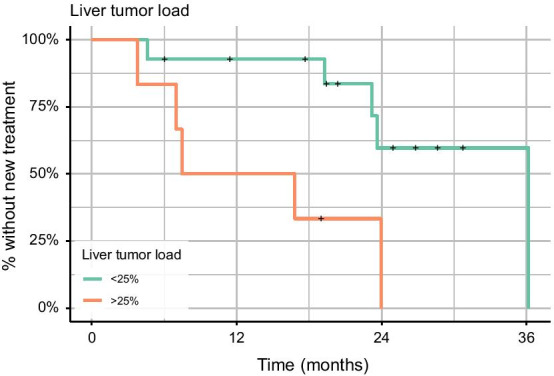
Liver tumor burden and time-to-new treatment. Relation between liver tumor burden and time-to-new treatment

Primary tumor, ECOG status, functional tumor, tumor grade, and baseline CgA level were neither associated with the amount of decrease in TL-SSTR or SSTR-TV, nor with time-to-new treatment, when tested individually. Liver tumor burden (i.e., > 25%) was associated with an increased hazard for time-to-new treatment (HR 5.17 [1.34–19.94], *p* = 0.02; Fig. 4). The percentual change in TL-SSTR and SSTR-TV was independent of baseline TL-SSTR or SSTR-TV values (*p* = 0.82 and *p* = 0.87).

## Discussion

Based on the presented data, the change in [^68^Ga]Ga-DOTA-TOC PET/CT parameters TL-SSTR and SSTR-TV does not significantly predict time to progression after PRRT. However, there seems to be a predictive value in both baseline and post-treatment TL-SSTR and SSTR-TV, though these parameters are strongly correlated.

Most patients show a notable decrease in both TL-SSTR and SSTR-TV after PRRT, independent of their baseline PET/CT characteristics or tumor characteristics. However, the magnitude of the change is often thought to be of importance in predicting the time to progression. Unfortunately, the current results do not provide conclusive evidence to support this assumption, due to lack of power. However, our data suggest that baseline and residual tumor volume after treatment is predictive for time-to-new treatment (*p* = 0.03 and *p* = 0.01), and that total post-treatment somatostatin receptor expression is predictive for time-to-new treatment (*p* = 0.04). Although nonsignificant in the current study with a small sample size, a trend exists for high pre-treatment TL-SSTR on [^68^Ga]Ga-DOTA-TOC PET/CT being a negative factor for time-to-new treatment (Fig. 2). However, this needs confirmation and validation in larger populations.

There is potential benefit to be gained from this increasingly used imaging technique during PRRT. As shown, high tumor volume on post-treatment imaging seems to be a potential negative predictor of time-to-new treatment. For example, in patients with a high tumor burden, a more stringent follow-up with close monitoring may be mandatory. More specifically, a high liver tumor burden was associated with earlier follow-up treatment. Thus, special attention should be paid to the treatment of liver tumors. Also, considering both pre-treatment tumor volume and liver tumor burden are associated with earlier time-to-new treatment, combined therapy of PRRT with radioembolization or with capecitabine/temozolomide could be considered in patients with a high overall tumor burden (Braat et al. 2020; Thakral et al. 2018).

It is known that pre-treatment [^68^Ga]Ga-DOTA-TOC PET/CT is useful for selecting patients for treatment with PRRT, and to predict response after PRRT (Toriihara et al. 2019; Sharma et al. 2019; Tirosh et al. 2018; Soydal et al. 2016; Kratochwil et al. 2015). Kratochwil et al*.* hypothesized that a high SUV_max_, and thus a high density SSTR-expression, would result in increased treatment response after PRRT. In a per-tumor analysis, they found a significant difference in pre-therapeutic tumor SUV_max_ between responding and non-responding lesions. However, no clear cutoff could be determined to select patients for PRRT, due to moderate sensitivity and specificity. This was confirmed by Sharma et al*.*, who found that a cutoff value for SUV_max_ of 13.0 provided a sensitivity of 0.83 and specificity of 0.84. Both studies showed that applying a cutoff value in selecting patients is a trade-off between treating patients that will not benefit from PRRT, and withholding treatment from patients that might benefit in terms of response. Besides [^68^Ga]Ga-DOTA-TOC PET/CT, [^64^Cu]Cu-DOTA-TATE PET/CT and [^18^F]FDG PET/CT have been shown to provide additional prognostic information in NEN patients (Carlsen et al. 2020; Zhang et al. 2020). Haug et al*.* demonstrated the feasibility of comparing the tumor-to-spleen SUV ratio between the [^68^Ga]Ga-DOTA-TATE PET/CT before PRRT and after one cycle (Haug et al. 2010). In 23/31 patients with decreased tumor-to-spleen SUV ratio after the first PRRT cycle, PFS was longer. Besides uptake and volume parameters, the utility of intra-tumoral imaging features on [^68^Ga]Ga-DOTA-TOC PET/CT was studied to predict response to treatment and overall survival, with promising results (Werner et al. 2017, 2019).

Considering the difficulty to predict response to PRRT and benefit of PRRT on survival before treatment, there is need for data on the predictive value of ^68^Ga-SSTR PET/CT pre- and post-PRRT. Unfortunately, these data are currently limited. No studies have been performed to assess the prognostic utility of a PET/CT-based response on PFS or clinical outcome. This study shows that the assessment of the studied changes on PET/CT is easy to acquire in clinical practice, due to the (semi-)automated delineation process. The method used in this study follows the PERCIST guidelines, and is thus easily reproducible and shown to be of clinical value in previous studies. Therefore, the true predictive value could be easily assessed in larger prospective studies.

This study has several limitations. First, the cohort size is small and heterogeneous. This is the cause for incoherent and not statistically significant results. However, the study shows the potential of the studied PET-parameters in standard acquired PET/CT to be used for outcome prediction. Furthermore, the current study population is rather heterogeneous, concerning tumor and patients characteristics. Most importantly, tumor grade can be a significant confounder, as it is related to both the studied PET-parameters and survival. In larger prospective cohort studies, this should be taken into consideration. In the current study, no subgroup analysis could be performed, and due to the infrequent occurrence of NET, further restrictions on inclusion criteria were not feasible. Secondly, due to the retrospective nature of the study, no predefined study protocol for long-term follow-up imaging was used. Objective radiological measurements for PFS (i.e., RECIST 1.1) in non-standardized follow-up are unreliable. However, the currently used outcome is clinically most relevant. Thirdly, uptake measurements were based on [^68^Ga]Ga-DOTA-TOC PET/CT, by which referral for additional treatment could also be driven. Hence, a bias is introduced. In our study however, only one patient was referred for treatment based on the follow-up [^68^Ga]Ga-DOTA-TOC PET/CT, so bias was limited. Finally, the used PET parameters, first used in [^18^F]FDG PET/CT, are not sufficiently validated as indicators of response. On the other hand, this does not prevent the use of these parameters in prediction of outcome. It is also important to note that TL-SSTR and SSTR-TV are closely related, given that they are both dependent on total tumor burden. Finally, the tumor volume (which is part of the TL-SSTR and SSTR-TV parameters) is probably not linked one to one to the biology of the neoplasms. Therefore, other parameters should always be taken into account, such as differentiation of the tumor, WHO grade, age, and staging of the disease.

To conclude, the current data show that automatically derived [^68^Ga]Ga-DOTA-TOC PET/CT parameters are easy to acquire, and may be of prognostic value after completing PRRT. Further studying of changes on [^68^Ga]Ga-DOTA-TOC PET/CT after PRRT should be done in larger cohorts, as it might have prognostic potential and help in patient planning.

## Data Availability

The data that support the findings of this study are available from the corresponding author upon reasonable request**.**
